# Reversible Lectin Binding to Glycan-Functionalized Graphene

**DOI:** 10.3390/ijms22136661

**Published:** 2021-06-22

**Authors:** Tereza Koukalová, Petr Kovaříček, Pavla Bojarová, Valentino L. P. Guerra, Vladimír Vrkoslav, Lukáš Navara, Ivan Jirka, Marek Cebecauer, Vladimír Křen, Martin Kalbáč

**Affiliations:** 1 J. Heyrovsky Institute of Physical Chemistry of the Czech Academy of Sciences, Dolejškova 2155/3, 182 23 Prague, Czech Republic; koukalot@vscht.cz (T.K.); guerrav@vscht.cz (V.L.P.G.); lukannavara@gmail.com (L.N.); ivan.jirka@jh-inst.cas.cz (I.J.); marek.cebecauer@jh-inst.cas.cz (M.C.); martin.kalbac@jh-inst.cas.cz (M.K.); 2 Department of Organic Chemistry, Faculty of Chemical Technology, University of Chemistry and Technology Prague, Technická 5, 166 28 Prague, Czech Republic; 3 Institute of Microbiology of the Czech Academy of Sciences, Vídeňská 1083, 142 20 Prague, Czech Republic; kren@biomed.cas.cz; 4 Department of Health Care Disciplines and Population Protection, Faculty of Biomedical Engineering, Czech Technical University in Prague, Nám. Sítná 3105, 272 01 Kladno, Czech Republic; 5 Institute of Organic Chemistry and Biochemistry of the Czech Academy of Sciences, Flemingovo Náměstí 542/2, 166 10 Praha, Czech Republic; vladimir.vrkoslav@uochb.cas.cz

**Keywords:** graphene, wheat germ agglutinin, carbohydrate, 2D materials, sensor

## Abstract

The monolayer character of two-dimensional materials predestines them for application as active layers of sensors. However, their inherent high sensitivity is always accompanied by a low selectivity. Chemical functionalization of two-dimensional materials has emerged as a promising way to overcome the selectivity issues. Here, we demonstrate efficient graphene functionalization with carbohydrate ligands—chitooligomers, which bind proteins of the lectin family with high selectivity. Successful grafting of a chitooligomer library was thoroughly characterized, and glycan binding to wheat germ agglutinin was studied by a series of methods. The results demonstrate that the protein quaternary structure remains intact after binding to the functionalized graphene, and that the lectin can be liberated from the surface by the addition of a binding competitor. The chemoenzymatic assay with a horseradish peroxidase conjugate also confirmed the intact catalytic properties of the enzyme. The present approach thus paves the way towards graphene-based sensors for carbohydrate–lectin binding.

## 1. Introduction

Two-dimensional (2D) materials hold a great potential for application as the active layers of sensors due to their strict monolayer character. Graphene, the epitomical example of a 2D family of materials, can achieve sensitivity down to single atoms under particular conditions [1]. However, the great sensitivity of graphene is compensated by its poor selectivity, which hampers the straightforward transfer of the technology to practice. The cornerstone of graphene-based sensor development is thus to achieve selective recognition of a given analyte [2,3].

Lectins are a broad family of proteins [4,5], featuring a carbohydrate recognition domain (CRD) that binds sugar moieties with a high specificity. Lectins play diverse roles in biological systems; they participate in cellular signaling, are involved in biochemical pathways leading to various pathologies (cancer [6], arthritis [7], etc.), and are essential in cell–cell recognition in infectious diseases such as AIDS [8,9], tuberculosis [10], and even the SARS-CoV-2 virus [11]. Lectins have been shown to form complex quaternary structures, ranging from dimers to higher homo- or heterooligomers up to chimeric structures that can gradually interchange [12,13]. This challenge is further complicated by the agglutinating properties of lectins and the chemical resemblance of their carbohydrate ligands.

Monolayer graphene functionalized with specific carbohydrate ligands can serve as a suitable active sensing layer for detecting lectin activity [14]. When placed into ionic solutions, it forms an electric double layer of ions on its surface [15,16,17,18]. Protein binding changes the Stern layer composition, which can be used as a functional principle of a sensor. In this way, highly specific graphene-based sensors of various lectin proteins can be constructed and used in biochemical and biomedical research and clinical practice. However, the major challenge of achieving the specific lectin–carbohydrate binding and distinguishing it from a non-specific interaction remains [19,20,21].

In this work, we demonstrate the specific binding of wheat germ agglutinin (WGA) to monolayer graphene covalently functionalized with *N*-acetylglucosamine (GlcNAc) chitooligomers of varying lengths. WGA is a 34 kDa homodimeric lectin with a total of eight carbohydrate binding sites [22], which features a high affinity to chitooligomers composed of several subunits of GlcNAc. We used a series of techniques [23] to characterize functionalization up to the reversible WGA binding in unprecedented detail. The data unambiguously confirm the scheme of chemical transformations taking place on the 2D monolayer. Ligand competition studies confirm the higher avidities of WGA for longer chitooligomers [24] and, therefore, the specificity of interactions on the functionalized surface, which is the crucial step in the development of graphene-based lectin sensors.

## 2. Materials and Methods

### 2.1. Synthesis of Functionalized Chitooligomers

**Scheme 1:**
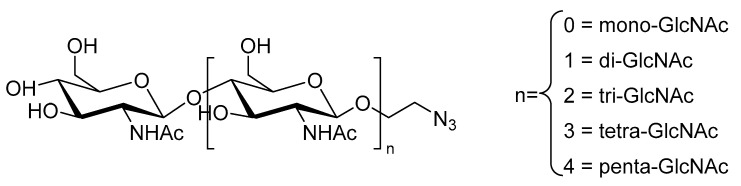
Structures of the GlcNAc chitooligomers used in this study.

For the preparation of 2-azidoethyl-functionalized chitooligosaccharides composed of β(1→4)-bound **GlcNAc** units (Scheme 1), we used a one-step transglycosylation reaction catalyzed by the Tyr470Asn mutant of the β-*N*-acetylhexosaminidase from *Talaromyces flavus*, which we had developed previously [25]. In this reaction, 2-azidoethyl 2-acetamido-2-deoxy-β-d-glucopyranoside (GlcNAc-*O*-EtN_3_; **mono-GlcNAc**), prepared chemically [24], was used as a glycosyl acceptor. 2-Azidoethyl 2-acetamido-2-deoxy-β-d-glucopyranosyl-(1→4)-2-acetamido-2-deoxy-β-d-glucopyranoside (**di-GlcNAc**), 2-azidoethyl 2-acetamido-2-deoxy-β-d-glucopyranosyl-(1→4)-2-acetamido-2-deoxy-β-d-glucopyranosyl-(1→4)-2-acetamido-2-deoxy-β-d-glucopyranoside (**tri-GlcNAc**), 2-azidoethyl 2-acetamido-2-deoxy-β-d-glucopyranosyl-(1→4)-2-acetamido-2-deoxy-β-d-glucopyranosyl-(1→4)-2-acetamido-2-deoxy-β-d-glucopyranosyl-(1→4)-2-acetamido-2-deoxy-β-d-glucopyranoside (**tetra-GlcNAc**), and 2-azidoethyl 2-acetamido-2-deoxy-β-d-glucopyranosyl-(1→4)-2-acetamido-2-deoxy-β-d-glucopyranosyl-(1→4)-2-acetamido-2-deoxy-β-d-glucopyranosyl-(1→4)-2-acetamido-2-deoxy-β-d-glucopyranosyl-(1→4)-2-acetamido-2-deoxy-β-d-glucopyranoside (**penta-GlcNAc**) were then prepared by transglycosylation reaction based on a previously reported procedure [24]. The structural characterization data (NMR, HRMS) of compounds **mono-** to **penta-GlcNAc** are fully in accordance with the literature [24].

### 2.2. Graphene Synthesis and Functionalization

Graphene was synthesized by the chemical vapor deposition method on a copper foil, transferred onto silicon chips with a 300 nm-thick SiO_2_ layer by the copper etching/polymer-assisted method, and fluorinated as described earlier [26,27]. The terminal alkyne was introduced by nucleophilic substitution of fluorine by propargyl amine deposited on the chip as a 50% solution in *N,N*-dimethylformamide at room temperature for two hours and then washed with a large excess of methanol.

### 2.3. The Cu-Catalyzed Alkyne-Azide (CuAAC) Reaction

**GlcNAc**-*O*-EtN_3_ ligand (2 mg) was dissolved in CuSO_4_ solution (900 µL, 100 µM) and mixed with sodium ascorbate solution (50 µL, 20 mM), and *tris*(3-hydroxypropyltriazolylmethyl)amine solution (50 µL, 6 mM)—all aqueous. This mixture was dropped onto the propargylated graphene chip (*ca* 1 × 1 cm^2^, *ca* 100 µL per chip) and reacted at room temperature. After 30 min, an additional 50 µL of 20 mM sodium ascorbate solution was dropped onto each chip and reacted for an additional 90 min. All samples were then thoroughly washed with deionized water and air dried.

### 2.4. Incubation with WGA and Competitor

Wheat germ agglutinin (WGA, from *Triticum vulgaris*, Sigma-Aldrich, St. Louis, MI, USA) was dissolved in phosphate-buffered saline (PBS) at an approximate concentration of 1 mg/mL, and 100 µL of this solution was dropped onto each chip (*ca* 1 × 1 cm^2^). Incubation was performed at room temperature for 2 h, and the samples were then washed with PBS buffer. Competitive unbinding of WGA was performed using **GlcNAc** solutions of defined concentrations in PBS at room temperature for 2 h followed by washing with PBS and deionized water.

## 3. Results and Discussion

**Figure 1:**
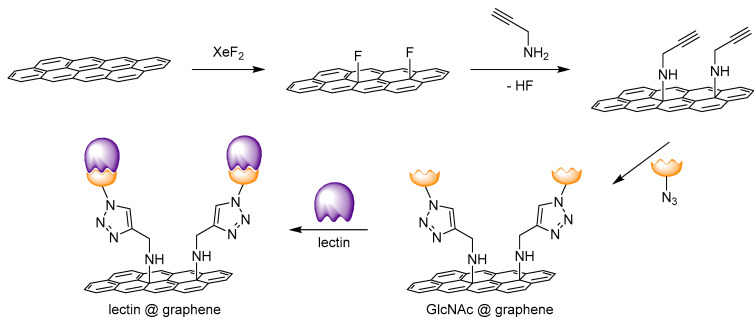
Scheme of the graphene chemical functionalization with chitooligomers for WGA binding. In the first step, pristine graphene was fluorinated, followed by nucleophilic exchange with propargyl amine. Using the CuAAC click reaction, azidoethyl-substituted chitooligomers (GlcNAc)_1–5_ were grafted on the functionalized graphene. WGA lectin binds to chitooligomer ligands immobilized on the graphene surface.

Monolayer graphene is rather inert and aggressive reagents are required for its activation (Figure 1). We used the previously reported fluorination with XeF_2_ in the gas phase at room temperature under reduced pressure [28,29]. The initial high fluorine content (ca 40 atomic % with respect to carbon) decreased within two days to an equilibrium value of about 10 atomic % [29], which means that every C–F bond is surrounded by nine sp^2^ carbon atoms on average. This arrangement renders the fluorine atom relatively easily exchangeable for suitable nucleophiles [28,30], such as the amino group of propargyl amine. Thus, the monolayer graphene may efficiently be grafted with terminal alkynes. In the next step, chitooligomers of different lengths carrying an azidoethyl substituent at the anomeric carbon were attached to the graphene surface using the standard CuAAC click reaction protocol in water [31]. Finally, specific binding between chitooligomer ligands and the corresponding lectin (WGA) occurred in aqueous PBS. The multistep functionalization scheme is shown in Figure 1.

**Figure 2:**
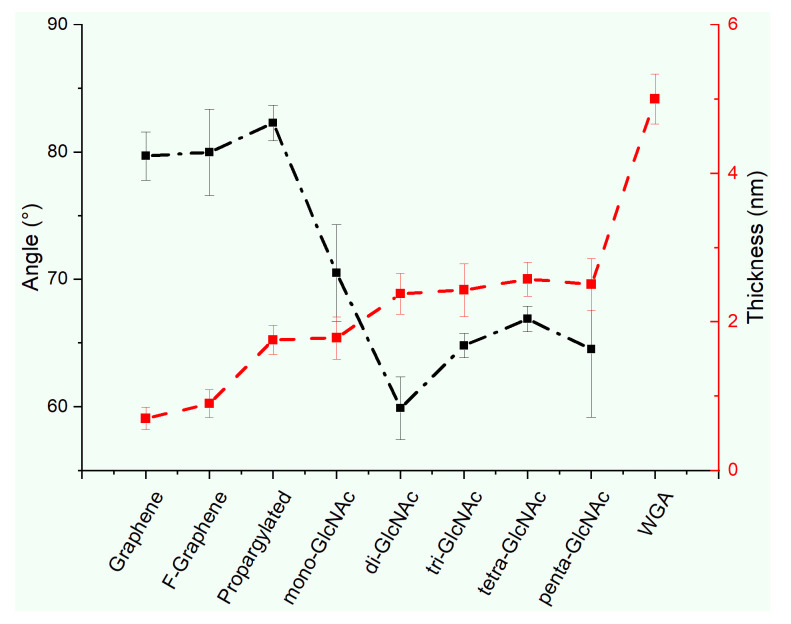
Summary of the AFM and CA data for graphene functionalization. AFM shows a gradual increase in the layer thickness with increasing size of the grafted species. Longer chitooligomers tended to fold back; hence, the thickness reached a plateau for tri- to penta-GlcNAc species. Importantly, after incubation with WGA, all chitooligomers showed a constant thickness of about 5 nm. Correspondingly, the CA decreased when hydrophilic carbohydrates were grafted onto the graphene surface, and a plateau of between 60° and 65° was reached for tri-GlcNAc and longer chitooligomers. See the Supplementary Materials for definitions of error bars.

The process of graphene functionalization was monitored after each step by several methods in order to unambiguously prove the successful grafting of the desired species according to the designed scheme. Graphene on the Si/SiO_2_ substrate is slightly hydrophilic [32,33], with a water contact angle (CA, Figure 2) of 79.7 (±1.9)°. Fluorination and propargylation led to negligible changes in the contact angle, with values approaching 80.0 (±3.4)° and 82.3 (±1.4)°, respectively. In contrast, a large difference in hydrophilicity was observed after the click reaction with chitooligomers—a single GlcNAc unit (mono-GlcNAc) decreased the contact angle to 70.5 (±3.8)°, while a di-GlcNAc ligand decreased it to 59.9 (±2.5)°. For longer chitooligomers, the CA value did not change any further and fell within a range of 60°–65°. Atomic force microscopy (AFM, Figure 2, see the Supplementary Materials file) confirmed the expected changes by providing complementary information on the layer thickness measured at the edge of the graphene sheet after each step. The pristine transferred graphene thickness was 0.7 nm. It increased to 0.9 nm after fluorination and to 1.7 nm after propargylation. Such an increase in thickness is in good agreement with the particular bond lengths (C–F, C–N, and C–C) in the grafted species^28^. After the reaction with chitooligomers, the layer thickness gradually increased along with the number of GlcNAc units, reaching a plateau at around 2.5 nm for the tri-GlcNAc ligand. Again, this value agrees with the CA values discussed above and the expected folding of longer flexible oligosaccharide chains on the surface. Importantly, AFM analysis of graphene decorated with the series of chitooligomers (from mono- to penta-GlcNAc) after incubation with WGA in PBS showed a uniform layer thickness of 5–6 nm, which is the estimated size of the 34 kDa globular WGA protein. These results thus support the hypothesis that chitooligomer ligands on graphene bind to WGA binding sites and also rule out the possibility of multiple-layer non-specific aggregation. The summary of CA and AFM data is provided in Figure 2.

**Figure 3:**
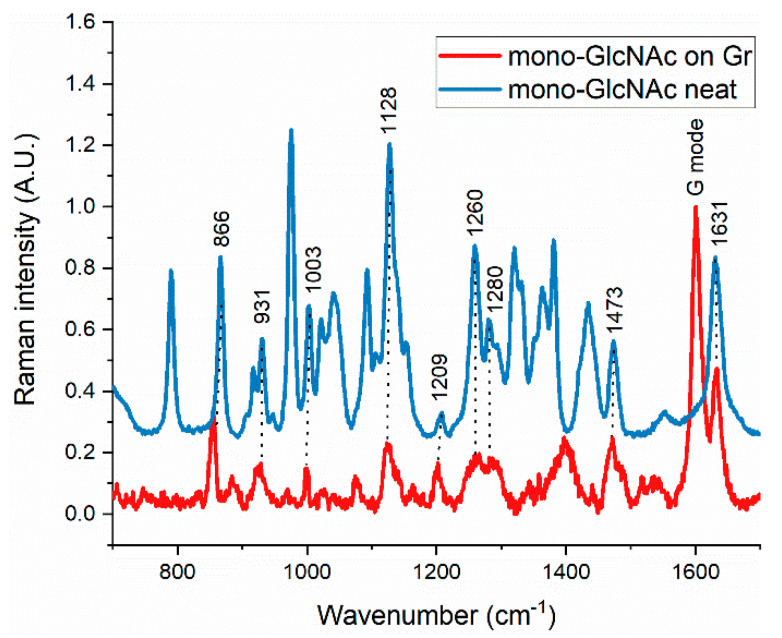
Comparison of Raman and surface-enhanced Raman spectra of mono-GlcNAc neat (blue) and grafted on graphene (red), respectively. Specific vibrations enhanced in the SERS mode are indicated in the spectra, e.g., amide I at 1631 cm^−1^, CH_2_ deformation at 1473 cm^−1^, and C–H and C–OH deformation at 1128 cm^−1^. The signal assignment was performed according to the literature [34,35].

The structural integrity of chitooligomers grafted to the surface was confirmed by surface-enhanced Raman spectroscopy (SERS, Figure 3). Reference spectra of the chitooligomers were measured in the solid state, showing sharp peaks at expected positions (partial assignment for mono-GlcNAc is given in the caption of Figure 3). With the increasing number of units, the bands broadened due to minor differences in the surrounding particular functional groups for longer chitooligomers, making the spectra non-informative for tri-, tetra-, and penta-GlcNAc ligands (see Supplementary Figure S1). Graphene samples after the click reaction were covered by a 12.5 nm-thick evaporated silver film, and Raman spectra were measured with a 633 nm excitation laser at intensities of 1–10 µW, focused with a 100× objective at a spot of about 1 µm. The laser intensity was carefully optimized because the thermal plasmon decay leads to overheating in the irradiated spot and to the degradation of the sample. The obtained SERS spectra matched well with the reference spectra, showing, for example, the characteristic amide I vibration at 1631 cm^−1^, CH_2_ deformation at 1473 cm^−1^, C–H and C–OH deformation at 1128 cm^−1^, and anomeric C–H deformation at 866 cm^−1^ (Figure 3, assignment according to the literature [34,35]). It is important to note that the enhancement of particular bands in SERS strongly depends on the orientation of the molecular vibration with respect to the plasmon field, as it has been shown previously [36]. Therefore, relative band intensities between conventional and surface-enhanced Raman spectra cannot be directly compared.

**Figure 4:**
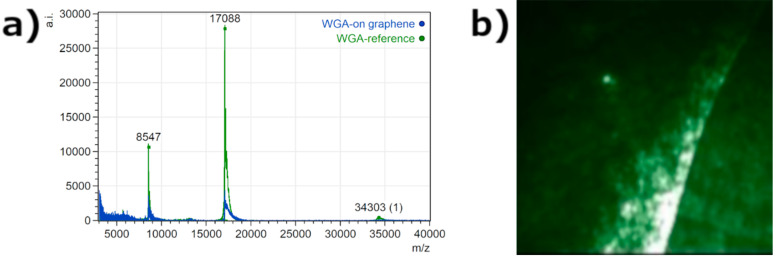
(**a**) MALDI spectra overlaying the reference spectrum of WGA (green), and the spectrum acquired on functionalized graphene after incubation with WGA (blue). The dominant masses in both spectra are 8.5, 17.1, and 34.3 kDa. (**b**) Fluorescence imaging of a graphene edge functionalized with GlcNAc after incubation with fluorescently labeled (fluorescein isothiocyanate, FITC) WGA. A clear contrast is observed at the edge of functionalized graphene.

To determine the interaction of lectin with chitooligomers, WGA was incubated with the GlcNAc-functionalized graphene for two hours at room temperature in PBS buffer. The successful binding was evaluated by matrix-assisted laser desorption/ionization mass spectrometry (MALDI) using sinapinic acid as the matrix. Functionalized graphene samples after incubation with either WGA and the reference WGA stock solution, deposited on silicon chips, both provided three dominant peaks at 8.5, 17.1, and 34.3 kDa (Figure 4a). The highest value corresponds to the natural WGA dimeric form, the middle value corresponds to the polypeptide monomer, and the lowest mass is the doubly charged monomer.

The MALDI results were further corroborated by fluorescence imaging. FITC-labeled WGA was incubated with functionalized graphene samples deposited on glass coverslips, excited using the 488 nm laser, and detected on a sensitive EM-CCD camera. Specific binding of FITC-WGA to GlcNAc-functionalized graphene was clearly detectable at the graphene edge as an intense contrast line (Figure 4b). Finally, the binding was confirmed by a chemoenzymatic assay. WGA conjugated with horseradish peroxidase (WGA-HRP, along with non-conjugated WGA as a negative control) was immersed into the tetramethylbenzidine (TMB) substrate solution. Within seconds, the characteristic blue color of the oxidized TMB developed in the sample with WGA-HRP but not in the negative control (see Supplementary Figure S3).

**Figure 5:**
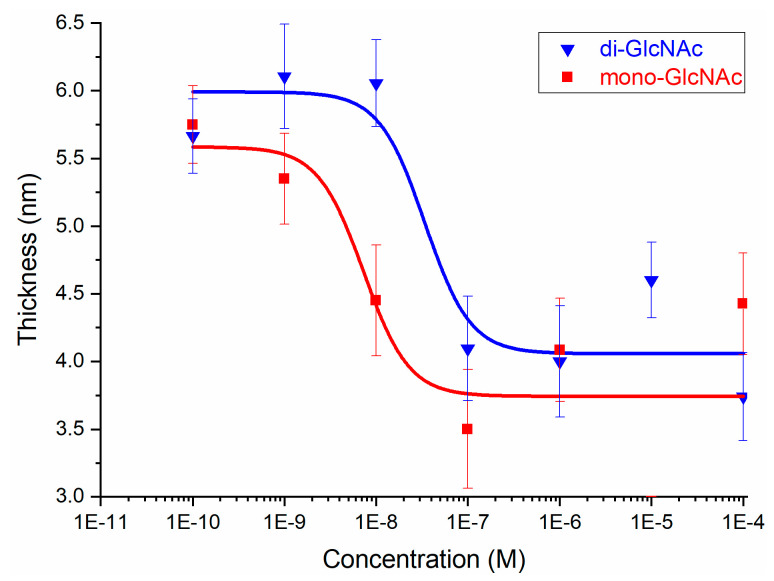
Comparison of the WGA binding curves obtained by AFM for mono- and **di-GlcNAc**-grafted graphene when in competition with free **mono-GlcNAc** ligand in solution. The curves were fitted with the Hill function with a fixed Hill coefficient of 1.75, as given in the literature [37]. The height drop for **di-GlcNAc**-grafted graphene occurred at about a five times higher competitor concentration than for the **mono-GlcNAc**-grafted graphene. The dependence of the dissociation constant on the chitooligomer length confirms the specificity of carbohydrate–lectin binding.

An important feature of carbohydrate binding to lectins is the previously shown affinity increase [24] by approximately one order of magnitude when extending the chitooligomer by a single GlcNAc unit. We thus performed a binding assay in which WGA bound to GlcNAc-functionalized graphene was competitively released from the surface by the addition of the monomeric ligand in the solution (see also Supplementary Figure S2). Figure 5 summarizes the AFM measurement of the layer thickness of graphene decorated with mono- or di-GlcNAc on the surface after competition with a gradually increasing concentration of mono-GlcNAc in solution. At low concentrations of the mono-GlcNAc competitor, the layer thickness showed constant values of 5.5–6 nm, indicating the presence of WGA bound to the surface. At a mono-GlcNAc concentration of about 10^−8^ M, the layer thickness suddenly dropped to values corresponding to the functionalized graphene without WGA. The physical nature of AFM measurement did not allow us to obtain experimental points in the descending slope of the sigmoidal curve. The experimental data were fitted with the Hill function with a fixed Hill coefficient of 1.75 (positive cooperative binding) based on previous studies^37^ and provided dissociation constants of 7.3 × 10^−9^ and 3.4 × 10^−8^ M for mono- and di-GlcNAc-functionalized graphene, respectively. For tri-GlcNAc and longer chitooligomers grafted on graphene, the competition with mono-GlcNAc did not proceed even at high competitor concentrations (10^−3^ M GlcNAc), indicating that mono-GlcNAc is not an efficient competitor for tri- and longer chitooligomers immobilized on the graphene surface.

## 4. Conclusions

Monolayer graphene was functionalized with β(1-4)-linked *N*-acetylglucosamine chitooligomers of varying lengths. Chitooligomers feature a high affinity for wheat germ agglutinin, a homodimeric lectin with eight carbohydrate binding sites. The successful functionalization of the graphene surface was confirmed by a set of complementary techniques including Raman, AFM, CA, SERS, fluorescence microscopy, and mass spectrometry. The critical issue of the binding specificity between lectin and chitooligomer ligands exposed on the graphene surface was investigated in a competitive binding assay using AFM as the monitoring technique. The extensive characterization consistently proved the mechanism of graphene functionalization and the specific binding of WGA to the carbohydrates grafted on the functionalized graphene. This study thus paves the way towards specific sensors for the quantitative determination of lectins, in diverse solutes, produced by industry or the natural environment.

## Data Availability

Not applicable.

## Supplementary Materials

The following are available online at https://www.mdpi.com/article/10.3390/ijms22136661/s1: Additional spectra, images, data tables, and instrumentation descriptions.
