# Supplementary material for: Reversible Lectin Binding to Glycan-Functionalized Graphene
Source: Int J Mol Sci. 2021 Jun 22;22(13):6661. doi: 10.3390/ijms22136661 (PMC8267698; doi:10.3390/ijms22136661)
Supplement: Supplementary file 1 [file ijms-22-06661-s001.zip › ijms-1257018-supplementary.pdf]

SUPPORTING INFORMATION

Reversible lectin binding to glycan-functionalized graphene

Tereza Koukalová,[1,2,3] Petr Kovaříček,[1,2,] Pavla Bojarová,[3,5,*] Valentino L. P. Guerra,[1,2] Vladimír Vrkoslav,[4] Lukáš Navara,[1] Ivan Jirka,[1] Marek Cebecauer,[1] Vladimír Křen,[3] Martin Kalbáč[1]*

[1] J. Heyrovsky Institute of Physical Chemistry of the Czech Academy of Sciences, Dolejškova 2155/3, 182 23 Prague, Czech Republic

[2] University of Chemistry and Technology Prague, Faculty of Chemical Technology, Dpt. of Organic Chemistry, Technická 5, 166 28 Prague, Czech Republic

[3] Institute of Microbiology of the Czech Academy of Sciences, Vídeňská 1083, 142 20 Prague, Czech Republic

[4] Institute of Organic Chemistry and Biochemistry of the Czech Academy of Sciences, Flemingovo náměstí 542/2, 166 10 Praha, Czech Republic

[5] Department of Health Care Disciplines and Population Protection, Faculty of Biomedical Engineering, Czech Technical University in Prague, Nám. Sítná 3105, CZ-27201 Kladno, Czech Republic

** petr.kovaricek@vscht.cz, bojarova@biomed.cas.cz*

Instrumentation

Water contact angle was measured by sessile drop technique with a setup from Advex Instruments. The drop of deionized water that was used had a volume of 0.5 μL; the contact angle was measured 30 seconds after it was deposited on the surface using an Eppendorf pipette (Eppendorf, Hamburg, Germany). Five drops of deionized water were used in each experiment and the observed values were averaged. Error bars are defined as standard deviation ofthe five measurements.

Samples for fluorescence imaging were prepared on round 25 mm coverslips (No 1.5H, High precision; Marienfeld). Graphene transfer and functionalization was described above.
Microscope setup and measurements. Fluorescence imaging was performed on a own-built microscope (IX71 body; Olympus) equipped with 150mW 488nm (Sapphire; Coherent) laser, 100x 1.49 NA objective (UApoN; Olympus) and EMCCD camera (iXon DU-897, Andor). Synchronisation of laser switching, and camera recording was performed with acousto-optic tuneable filter (AOTF; AOTFnC-400.650-TN, AA Optoelectronics) and an own-written acquisition software (LabView). Acquisition time was 100 ms/frame.
Data analysis. Minor adjustments to compensate the optical field profile were applied to images using the ImageJ/Fiji software package.

The Raman spectra were measured with a WITec Alpha300 R spectrometer equipped with a piezo stage with 532 nm excitation laser wavelengths. The laser was focused on the sample with a 100× objective to a spot with a diameter of <1 μm. SERS spectra were acquired using a LabRAM HR (HORIBA Jobin-Yvon),

with Olympus BX microscope, 100x vis LWD lenses, excited by 633 nm wavelength. Silver film evaporation was performed using a picoSphere instrument (Oxford Vacuum Science).

The AFM images were measured with the Bruker Dimension Icon using silicon probes for soft TappingMode imaging (RFESPA-75). The measurements were performed in the PeakForce tapping mode on 10 x 10 μm^2, with the resolution adapted for every image (512 lines), and a scanning rate of 0.4 Hz. The images were further processed using the Gwyddion software [38]. Root mean square (RMS) roughness of the graphene sheet (along a 1 µm line) was taken as the error bar value (see Figure S 2).

MALDI experiments were performed on ultrafleXtreme (Bruker Daltonik GmbH, Bremen, Germany) operated in the positive linear mode and an acceleration voltage of 25 kV. The instrument is equipped by Smartbeam-II Nd:YAG UV laser (frequency of 1000 Hz; 355 nm). Samples of modified graphene on silicon substrate were attached to a TLC target plate (MTP TLC Adapter), using double-sided adhesive conductive tape. The data were collected in the m/z range of 3-40 kDa and analysed using the FlexAnalysis 3.3 software (Bruker Daltonik GmbH, Bremen, Germany) and mMass v5.5 [39–41]. The spectra were averaged from 8000 laser shots (8 x 1000 shots) collected in various places across the sample. External calibration using protein mixture (Protein calibration standard I, Bruker Daltonik GmbH, Bremen, Germany) deposited on silicon substrate was performed before measurement... Sinapinic acid was used as the ionization matrix (saturated solution in acetonitrile/water 1:1 acidified with 0.1 % TFA).

Additional experimental data

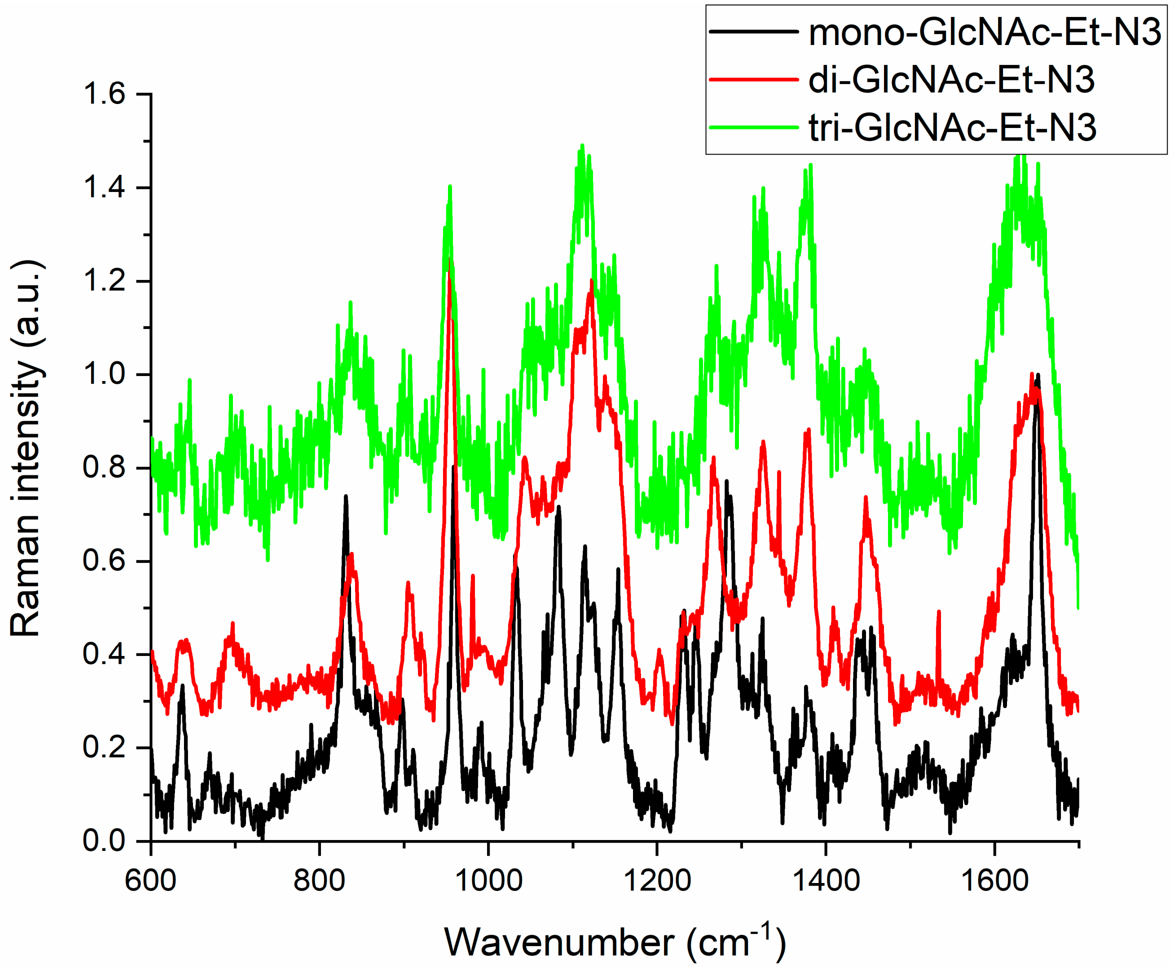

Figure S 1 Raman spectra of neat solids of azidoethyl-terminated chitooligomers. Mono-GlcNAc (black) provided well resolved bands which are in full agreement with the previously reported data. With additional units in the chitooligomer chain (di-GlcNAc in red , and tri-GlcNAc in green), the bands broadened and became generally non-informative for chitooligomers longer than three GlcNAc units.

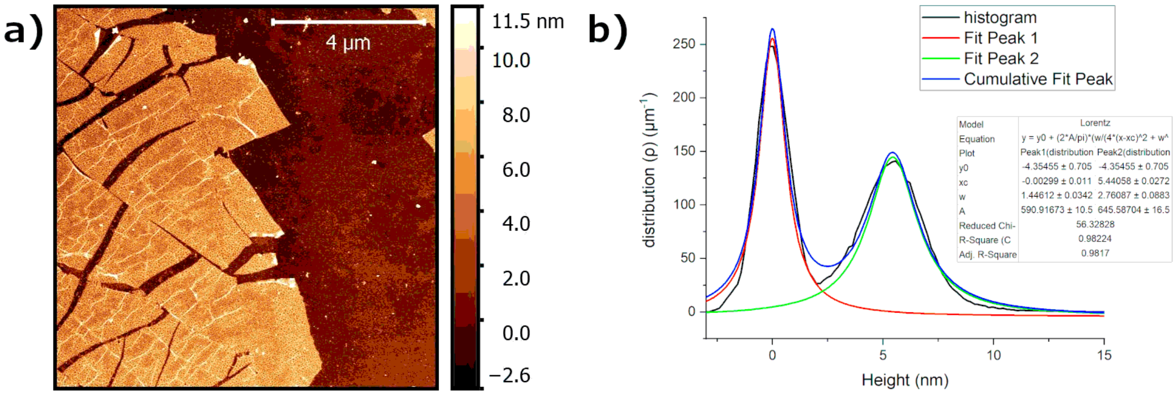

Figure S 2 Example of an AFM image and data processing used to obtain the sample thickness of samples shown in the main text. a) Image of the scanned 10 x 10 µm^2 area showing the edge of graphene sheet. Root mean square roughness of the graphene sheet (along a 1 µm line) was taken as the value for the error bar. b) Histogram of graphene height (black line) calculated from the whole image (512 x 512 points) was fitted with two Lorentz functions (red and green lines). The distance between the centers of the two peaks was taken as the layer thickness. The error of fitting was in all cases in the range of several tens of pm, which is more than one order of magnitude lower than the error determined from RMS roughness.

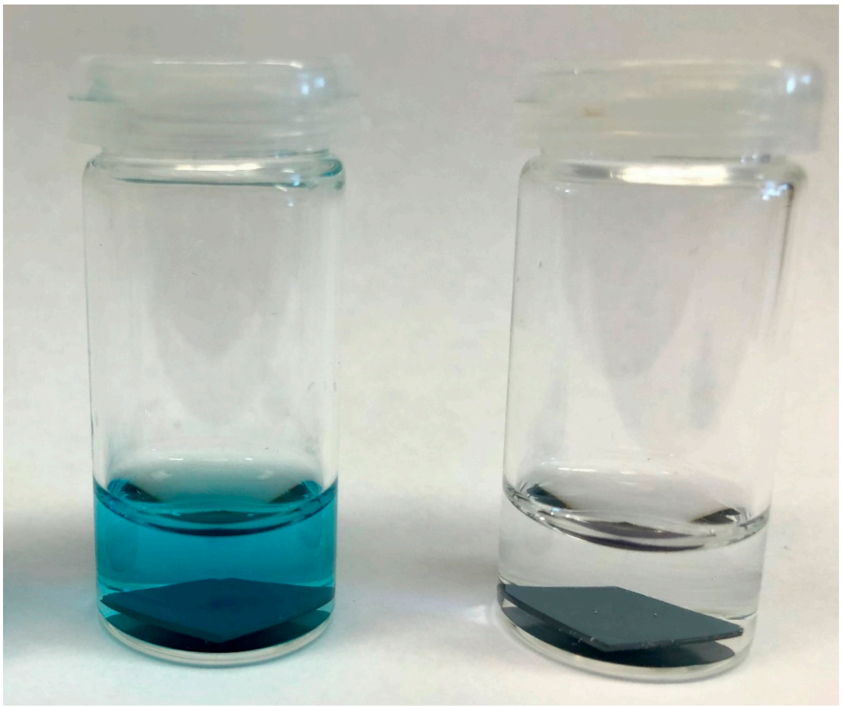

Figure S 3 Chemoenzymatic assay of protein activity after binding to graphene. WGA conjugated with horse radish peroxidase (HRP, left) and unlabelled standard WGA (right) were immersed into the TMB substrate (Sigma Aldrich). The blue color developed in the left vial demonstrates that WGA-HRP preserved its reactivity even upon binding to chitooligomer-grafted graphene (here, grafted with mono-GlcNAc ligand).

References

38. Nečas, D.; Klapetek, P. Gwyddion: An Open-Source Software for SPM Data Analysis. *Cent. Eur. J. Phys.* **2012**, *10*, 181–188, doi:10.2478/s11534-011-0096-2.

39. Niedermeyer, T.H.J.; Strohalm, M. MMass as a Software Tool for the Annotation of Cyclic Peptide Tandem Mass Spectra. *PLOS ONE* **2012**, *7*, e44913, doi:10.1371/journal.pone.0044913.
40. Strohalm, M.; Kavan, D.; Novák, P.; Volný, M.; Havlíček, V. MMass 3: A Cross-Platform Software Environment for Precise Analysis of Mass Spectrometric Data. *Anal. Chem.* **2010**, *82*, 4648–4651, doi:10.1021/ac100818g.
41. Strohalm, M.; Hassman, M.; Košata, B.; Kodíček, M. MMass Data Miner: An Open Source Alternative for Mass Spectrometric Data Analysis. *Rapid Commun. Mass Spectrom.* **2008**, *22*, 905–908, doi:10.1002/rcm.3444.
